# Bioelectronics-on-a-chip for cardio myoblast proliferation enhancement using electric field stimulation

**DOI:** 10.1186/s40824-020-00195-2

**Published:** 2020-09-07

**Authors:** Ángel Aragón, María Cebro-Márquez, Eliseo Perez, Antonio Pazos, Ricardo Lage, José Ramón González-Juanatey, Isabel Moscoso, Carmen Bao-Varela, Daniel Nieto

**Affiliations:** 1 Photonics4life group, Faculty of Physics, University of Santiago de Compostela, 15782 Santiago de Compostela, Spain; 2 Cardiology Group, Center for Research in Molecular Medicine and Chronic Diseases (CIMUS), University of Santiago de Compostela and Health Research Institute, University Clinical Hospital of Santiago de Compostela, Santiago de Compostela, Spain; 3 Department of Particle Physics, University of Santiago de Compostela, E-15782 Santiago de Compostela (A Coruña), Campus Sur Spain

**Keywords:** Cell electrostimulation, Bioelectronics chip, Laser microfabrication, Cell culture

## Abstract

**Background:**

Cardio myoblast generation from conventional approaches is laborious and time-consuming. We present a bioelectronics on-a-chip for stimulating cells cardio myoblast proliferation during culture.

**Method:**

The bioelectronics chip fabrication methodology involves two different process. In the first step, an aluminum layer of 200 nm is deposited over a soda-lime glass substrate using physical vapor deposition and selectively removed using a Q-switched Nd:YVO_4_ laser to create the electric tracks. To perform the experiments, we developed a biochip composed of a cell culture chamber fabricated with polydimethylsiloxane (PDMS) with a glass coverslip or a cell culture dish placed over the electric circuit tracks. By using such a glass cover slip or cell culture dish we avoid any toxic reactions caused by electrodes in the culture or may be degraded by electrochemical reactions with the cell medium, which is crucial to determine the effective cell-device coupling.

**Results:**

The chip was used to study the effect of electric field stimulation of Rat ventricular cardiomyoblasts cells (H9c2). Results shows a remarkable increase in the number of H9c2 cells for the stimulated samples, where after 72 h the cell density double the cell density of control samples.

**Conclusions:**

Cell proliferation of Rat ventricular cardiomyoblasts cells (H9c2) using the bioelectronics-on-a-chip was enhanced upon the electrical stimulation. The dependence on the geometrical characteristics of the electric circuit on the peak value and homogeneity of the electric field generated are analyzed and proper parameters to ensure a homogeneous electric field at the cell culture chamber are obtained. It can also be observed a high dependence of the electric field on the geometry of the electrostimulator circuit tracks and envisage the potential applications on electrophysiology studies, monitoring and modulate cellular behavior through the application of electric fields.

## Background

The application of electrical stimulation (ES) to cells culture to influence cell proliferation has been investigated as a possible method of treatment in several diseases [1–5]. ES is also used in biomedical research by the application of electrical signals in cell cultures for simulating different body conditions [6–9]. Applications can range from studying the growth and information processing of neurons [10] or the effect of electric impulses in cardiac cells [11], capillary electrophoresis chips for the separation of biochemicals such as amino acids and nucleotides [12] up microstructures for the analysis of DNA [13]. Mostly, such electrical stimulators present the same assembly that consist of two electrodes which are directly in contact with cells and culture medium and the electric impulse is externally applied [14–17]. This condition is not always appropriate because the electrodes may cause toxic reactions in the culture or may be degraded by electrochemical reactions with the cell medium [18, 19], so the design of the device will strongly depends on these restrictions.

The most sophisticated electrical stimulators use microcircuits instead of simple electrodes that in most of cases are fabricated using standard chemical bath or photolithography [20–22]. The main advantage of photolithography is its versatility, since allows the fabrication of microelectrodes with a broad range of shapes and sizes, nevertheless, it requires an elaborate work in various steps involving several chemical substances that can last about 3 h [23] to successfully fabricate the electrodes. As an alternative, selective laser elimination of thin materials deposited over glass substrates present some advantages in terms of time and material by being a relatively accessible and non-expensive technique [24–28]. While photolithography needs hours to fully fabricate the electrostimulator circuit, laser ablation can fully mark the same surface in a few minutes. Moreover, once you have a thin film of material only the laser interaction is needed to fabricate the electrode, instead of the chemical components. This technique uses the process of laser ablation, where the interaction of the laser energy with the sample leads to material removal. Usually this phenomenon depends on the absorption of laser photons by the sample material, which means that the wavelength of the laser should be chosen carefully for maximum absorption. However, the use of ultrafast lasers avoids this approach since ablation takes place as result of multi-photon absorption at high peak intensities, which means that even materials normally transparent to the laser wavelength can be processed [29].

In this paper we present a bioelectronics-on-a-chip for cardiomyoblasts cells proliferation enhancement using electrical stimulation. The electrical stimulator was fabricated using a laser-based fabrication technique. First, a 200 nm aluminum film was deposited over a soda-lime glass by physical vapor deposition (PVD). By using laser techniques, the aluminum film was selective removed to obtain a predesigned electric circuit which was able to apply electric stimulus in an area delimited by a polydimethylsiloxane (PDMS) layer over the circuit. To avoid toxic reactions in the culture or degradation by electrochemical reactions with the cell medium, a 145 μm glass was placed between the electric tracks and the culture medium. The electrical stimulator was then assembled between two polycarbonate layers to maintain the device compact and was used to study the effect of electrical stimulation of Rat ventricular cardiomyoblasts cells (H9c2). Since primary cardiomyocytes do not proliferate, we have chosen H9c2 cells as an alternative to primary cardiomyocytes; these cells maintain morphological characteristics of immature embryonic cardiomyocytes with electrical and hormonal signal pathway elements of adult cardiac cells. These cells have been extensively used in cardiovascular research and specifically in other electric stimulation assays [30–34].

Results shown a high dependence of the electric field on the geometry of the electrostimulator circuit tracks and envisage the potential applications on enhancement cell proliferation using electric field. For example, injection of serum cultured autologous myoblasts associated with coronary revascularization is a safe and feasible procedure and is associated with an increase in the myocardial viability index in the infarcted region and an improvement in left ventricular function. In this sense, the bioelectronics chip developed in this work emerge as powerful tool a to increase cardio myoblast cell production in vitro. Section 2 presents material and methods. Section 3 is devoted to results and Section 4 to conclusions.

## Materials and methods

### Laser set-up

A Nd:YVO_4_ Q-Switched pulsed laser (Power Line 20E, ROFIN-SINAR laser, Munich) operating at the fundamental wavelength of 1064 nm and with pulse width of 20 ns was used for fabricating the electric tracks. The laser beam was focused with a lens of 100 mm focal distance providing a uniform irradiance distribution in an area of 80 × 80 mm^2^ with a spot size at focus of 15 μm. The laser system is equipped with a mirror galvanometer system and a CAD-like software that allows drawing and defining the laser irradiation spatial distribution per unit time.

### Electric circuit materials

The glass used as a substrate was a commercial microscope slide of 26 × 76 mm, provided by Labbox (Labbox Labware, Barcelona, Spain). The material used for the thin layer deposition was aluminum with a purity of 99.98% and for fabricating the culture chamber in the first prototype we used polydimethylsiloxane, Silgard 184 (Dow Corning® 184 Silicone Elastomer, Michigan, USA).

### Thin layer deposition

Aluminum layers of 2019 Å of thick were deposited over soda lime glass substrates by physical vapor deposition (PVD) using a Balzers BAE 250 coating system (Oerlikon Balzers, Liechtenstein). In order to achieve a high-quality film, a three-steps cleaning process of the substrate is need. In all the steps an ultrasonic cleaner (Branson 5200, Danbury, USA) was used. At the beginning of the cleaning process samples were brush-scrubbed and then immersed in a soapy bath at a temperature of 35 °C for 30 min. After that, glasses were rinsed and bathed again with deionized water in the same conditions. Finally, they undergo a new ultrasound bath immersed in isopropyl alcohol (35 °C for 30 min) and were dried using pressurized air.

### Cell culture

Rat ventricular cardiomyoblasts cells (H9c2) (ATCC, Manassas, VA, USA) in 0.1% gelatin with DMEM (Sigma-Aldrich, St. Louis, MO, USA) medium supplemented with 10% fetal bovine serum (FBS) (Sigma-Aldrich, St. Louis, MO, USA), antibiotics (100 UI/mL penicillin, 100 μl/mL streptomycin) (Sigma-Aldrich, St. Louis, MO, USA) and 2 mM L-glutamine (Sigma-Aldrich, St. Louis, MO, USA), were maintained and stimulated in a 5% CO2 atmosphere at 37 °C. H9c2 cells were seeded at least 24 h before stimulation on cell imaging dishes (Eppendorf, Hamburg, Germany). After the electrical exposure, the cells were marked with the fluorescence stain DAPI (4′,6-diamidino-2-phenylindole) (Abcam, Cambridge, UK). In order to apply it, immediately after the experiments the cells are subjected to a fixation process with methanol at 20 °C for 15 min and then stored at 4 °C. The next step consists in permeabilize the cell membrane with 0.1% Triton X-100 (Sigma-Aldrich, St. Louis, MO, USA) at room temperature for 10 min and after that a 1% bovine serum albumin (BSA) solution (Sigma-Aldrich, St. Louis, MO, USA) at room temperature for 15 min. Finally, the DAPI marker is applied to the cell culture.

### Characterization methods

The thin layer deposited samples were examined using both optical and confocal microscopes. The optical microscope Nikon MM-400 (Nikon Metrology, Brighton, USA) was used to visualize the sample and predetermine damage at the surface. Morphological observations of the samples were undertaken by means of a Carl Zeiss Ultra Plus field emission scanning electron microscope (Zeiss, Oberkochen, Germany). Both the surface and the cross section after coating fracture were evaluated. The conductivity of the samples was determined via four-probe method. An electric current (5–30 mA) was passed through collinear outer metal electrodes by a Keithley 2400 source meter (Keithley Instruments, Cleveland, USA) and the voltage drop was measured between two inner electrodes with a HP 34401 A multimeter (Agilent Technologies, Santa Clara, USA). Considering that the distance between adjacent electrodes (s) was 2.5 mm and the thickness (t) of the films was close to 200 nm, the necessary condition for employing four-point probe method for conductivity measurements (t < <s) was satisfied. Once the electric circuit was fabricated, the electric impulses were applied by the NI USB-6501 portable digital I/O device (National Instruments, Austin, USA), which provides 5 V by default and up to 8.5 mA. For controlling this device, a software programmed in LabVIEW was used to apply a square signal whose parameters can be chosen by the user. The fluorescence microscopy images were obtained using the confocal microscope Leica TSC SP8 (Leica Microsystems CMS GmbH, Mannheim, Germany). To simulate the intensity and the homogeneity of the electric field above the electrical stimulator ANSYS Maxwell (ANSYS, Canonsburg, USA) was used. Circuits with different geometries were simulated and the electric field in different planes was estimated.

## Results

### Thin film deposition process

**Fig. 1:**
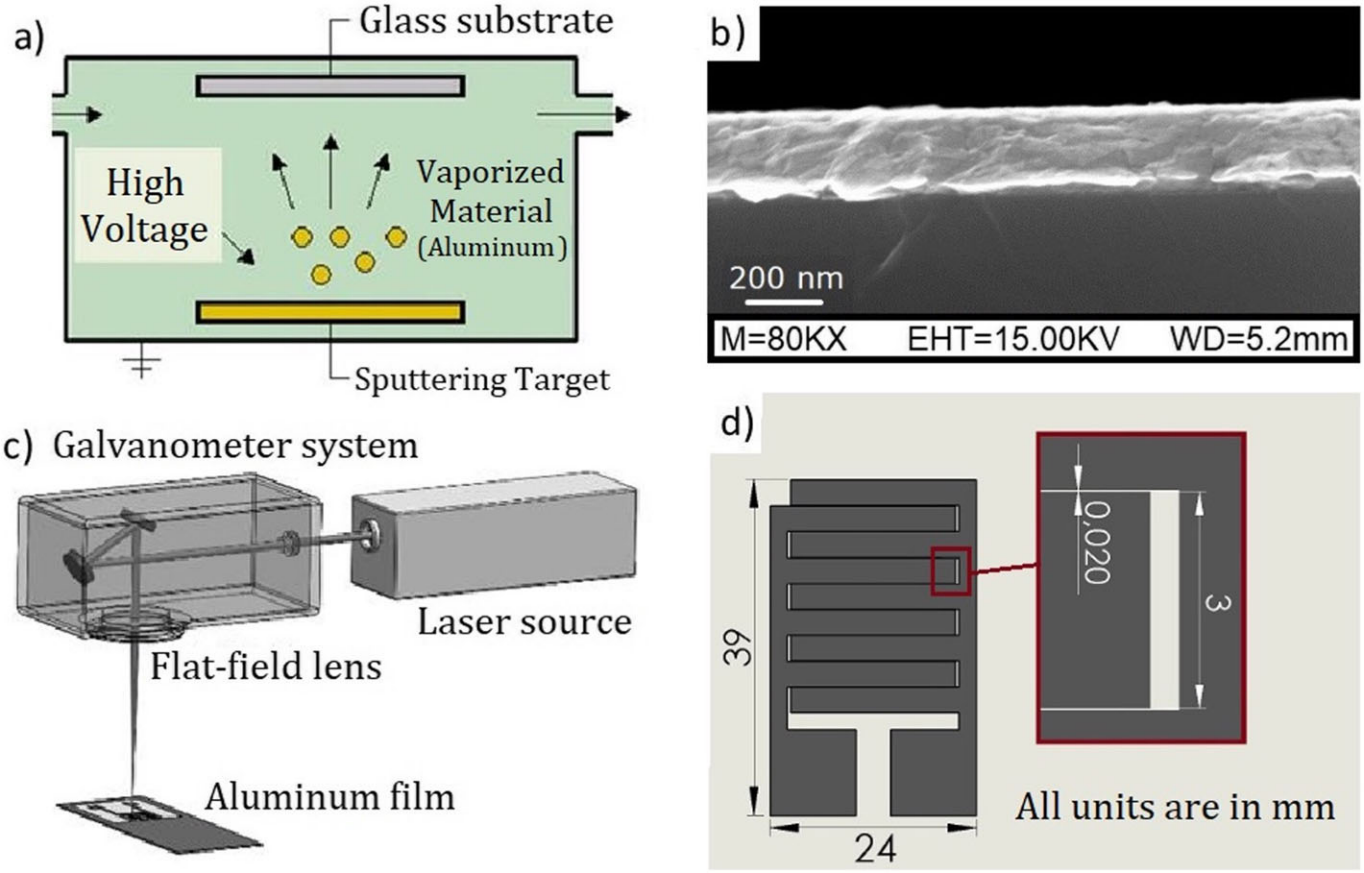
**a** Sketch of the PVD process for aluminum layer deposition, **b** image of the cross section of the aluminum films over the glass substrate after the thermal treatment. **c** Laser set-up for the ablation process; **d** CAD design of the electrostimulator with the zoomed view of the tracks design

Aluminum layers of 2019 Å over microscope slides were obtained by PVD, Fig. 1a shows a sketch of this technique. After the cleaning process described in section 2.3, glasses were introduced in the coating system where the aluminum layer was deposited at a deposition rate of 50 Å/s under a vacuum of 2 × 10^− 5^ mbar.

After that, the metalized substrate undergoes an annealing process at 200 °C for 2 h in an oven, that notably enhances the quality of the soldering afterwards and homogenize the coating surface [28]. Images of the profile of the layer have been taken by measuring the cross section of the samples with Scanning Electron Microscope SEM (Fig. 1b). Before the annealing processes, the layer presents a roughened surface with frequents clusters of particles. However, the thermal annealing leads to a smoother surface. In this case, the image was taken with a working distance of 5.2 mm, an extra high tension (EHT) of 15 KV and 8 × 10^4^ magnification.

Electric conductivity of the samples was measured before and after the thermal treatment obtaining that before the thermal treatment the electric conductivity of the layer is 2.250 ± 0.085 × 10^5^ S/cm while after the annealing process, the conductivity is 2.492 ± 0.086 × 10^5^ S/cm. Taking into account that the conductivity of the aluminum is 3.77 × 10^5^ S/cm, it can be asserted that it is reduced in a 40% due to the deposition process. However, it can be increased carrying out the annealing process.

### Laser fabrication of electrical tracks

The fabrication of the electrical stimulator circuit is based on the laser ablation technique. Aluminum layers were irradiated with a quasi-perpendicular nanosecond Nd:YVO_4_ laser emitting at a wavelength of 1064 nm. The laser is fitted with a galvanometer beam steering system and a flat-field lens of 100 mm focal distance (Fig. 1.d). This lens allows scanning the substrate within the X, Y plane. In this technique, the laser is focused onto the surface of aluminum layer generating an intense plasma plume that pull off Al ions and particles from the layer without causing any cracks on substrate (glass) surface. A specific pattern structure, generated by CAD-like software, was designed, and fabricated selectively removing part of the aluminum layers deposited as described in section 3.1.

Figure 1d, shows the CAD design used in electrical stimulator fabrication. Laser parameters setting were optimized in order to perform the metal layer removal without damaging the substrate. An average laser power of 1.05 W, a repetition rate of 12 kHz and a scan speed of 60 mm/s were selected to perform the ablation process. The aluminum was successfully removed using a laser fluence value between the damage threshold of glass and the ablation threshold of the target (920 J/cm^2^ and 4.20 J/cm^2^, respectively).

To determine the threshold fluence value for the aluminum layer, we follow the method of Liu [35].. Assuming an output gaussian beam for the laser, the spatial fluence (*φ*(*r*)) is given by

1 $$ \varphi (r)={\varphi}_0{e}^{\raisebox{1ex}{$-2{r}^2$}\!\left/ \!\raisebox{-1ex}{${\omega}_0^2$}\right.} $$

where ω_0_ is the gaussian beam radius (measured at 1/e^2^), *φ*_0_ is the peak fluence of the laser and r is the distance from the center of the beam. The energy per pulse (*E*_*pp*_) and the peak fluence are related according to the equation

2 $$ {\varphi}_0=\frac{2{E}_{pp}}{\pi {\omega}_0^2} $$

The relation between peak fluence and the diameter of the laser ablated spot (*D*) is given by

3 $$ {D}^2=2{\omega}_0^2\mathit{\ln}\left(\frac{\varphi_0}{\varphi_{th}}\right) $$

Combining eqs. 2 and 3 following relation is obtained

4 $$ {D}^2=2{\omega}_0^2\mathit{\ln}\left({E}_{pp}\right)-2{\omega}_0^2\mathit{\ln}\left(\frac{2}{\pi {\omega}_0^2{\varphi}_{th}}\right) $$

**Fig. 2:**
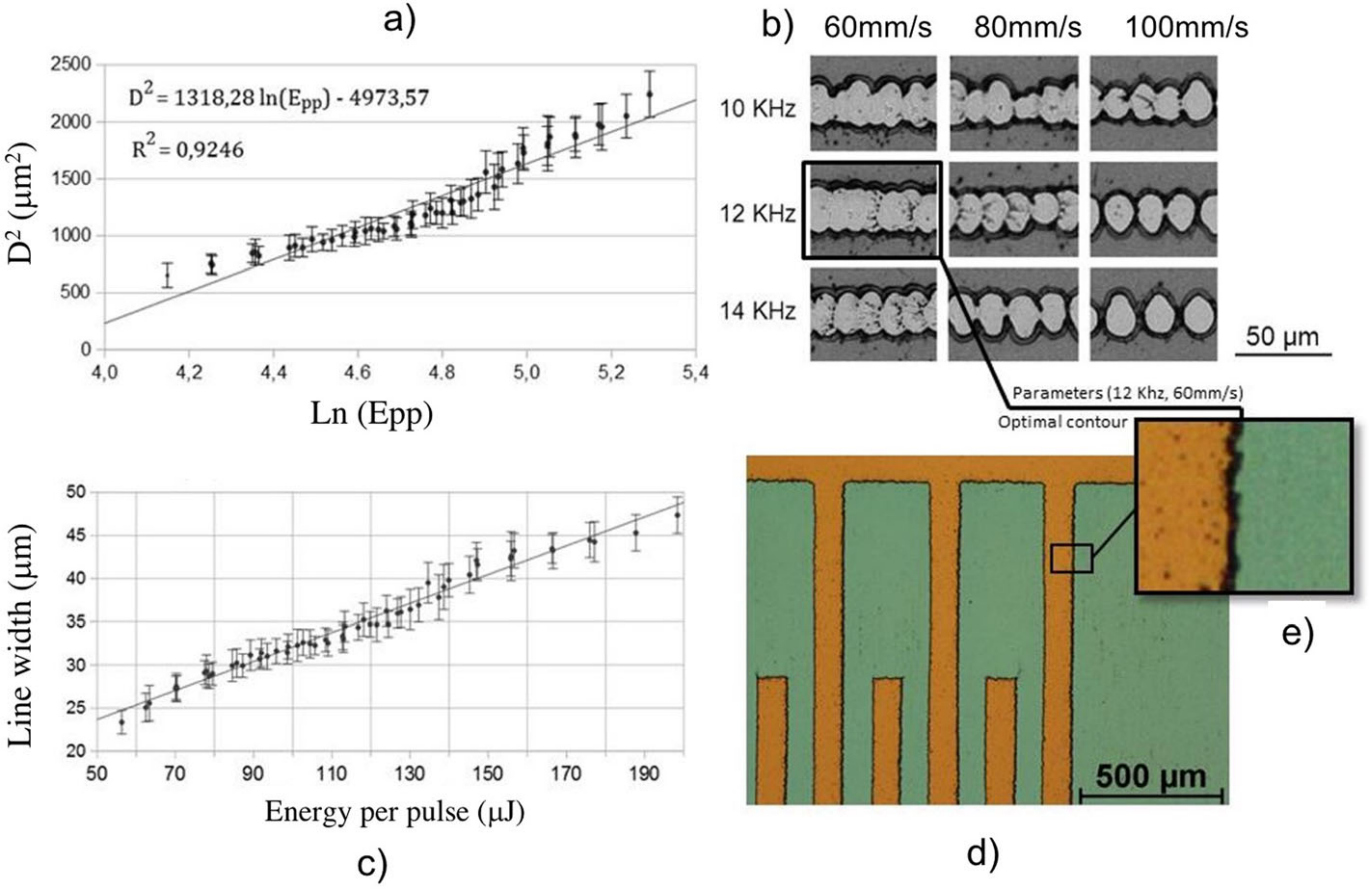
**a** Representation of *D*^2^ (diameter of the ablated spot) versus the logarithm of the energy per pulse over an aluminum layer. **b** Results of laser ablation in aluminum layers with different parameters. **c** Relation between the diameter of the laser mark and the energy per laser pulse. **d** Optical microscope photography of the tracks of one of the electrostimulator circuits fabricated

Therefore, using above equation threshold fluence and Gaussian beam spot size can be obtained by measuring the diameters of the ablated areas D. Plotting *D*^2^ versus the ln (E_pp_) and fitting the data, ω_0_ is determined and the threshold fluence is obtained extrapolating to D^2^ = 0. Results are shown in Fig. 2a, where values of 4.19 ± 0.77 J/cm^2^ and 25.69 ± 0.51 μm were obtained for aluminum layer threshold fluence and for the radius of the beam, respectively.

The proper selection of the laser parameters allows us to ablate the aluminum in the most efficient way without causing damage in the glass substrate. The adjustable parameters of the system used are the laser fluence, the laser frequency and the scan speed of the beam. To determinate the ratio between the laser frequency and the scan speed we define the degree of pulse overlap between consecutive spots. This factor is set in the equation

5 $$ {O}_d=1-\frac{v}{2 df} $$

where *v* and *f* are the scan speed and frequency, respectively, and *d* is the diameter of the spot crater. Pulse overlapping is a crucial parameter for fabricating a homogeneous electrical track. Excessive overlap will deliver too much energy to the glass, damaging the surface and increasing manufacturing time, while low overlap will result in inefficient material removal.

In Fig. 2b electrical tracks of a 200 nm aluminum layer ablated with different laser frequencies and scan speeds and therefore, with different pulse overlapping are presented. All of them were ablated with an average power of 700 mW. Results show how the ratio of 12 kHz and 60 mm/s produces tracks with more regular width. This correspond to an overlap degree of 0.66. In other cases, the pulses are either too separated or too overlapped. Based on these results, electrical tracks were fabricated using an overlapping factor of 0.66.

Finally, the optimal ratio between laser power and frequency was also analyzed. Tracks were fabricated with different values of both parameters. Figure 2c shows the diameter of a single line versus the energy per pulse, obtained by using different combinations of frequency (from 8 to 18 kHz) and power (from 700 to 2000 mW). It can be observed the linear relationship between the width of the line and the energy per pulse. This width was considered during the aluminum layer removal process. Therefore, the optimum laser parameters setting for the electrostimulator fabrication were, a pulse energy of 90 μJ (corresponding to a frequency of 12 kHz and a laser power of 1.05 W) combined with a scan speed of 60 mm/s.

In Fig. 2d, it can be observed an optical microscope image of a part of the electrical stimulator. The microscope is equipped with a double lighting system that allows illuminating samples both from above and below. In the Fig. 2d, orange region corresponds to the glass substrate and the green one to the aluminum tracks. Note that the image was taken lighting the electrical stimulator from below. The aluminum was successfully removed and there are no contacts between the tracks. Besides, the glass substrate was not damaged.

### Electrical tracks characterization

**Fig. 3:**
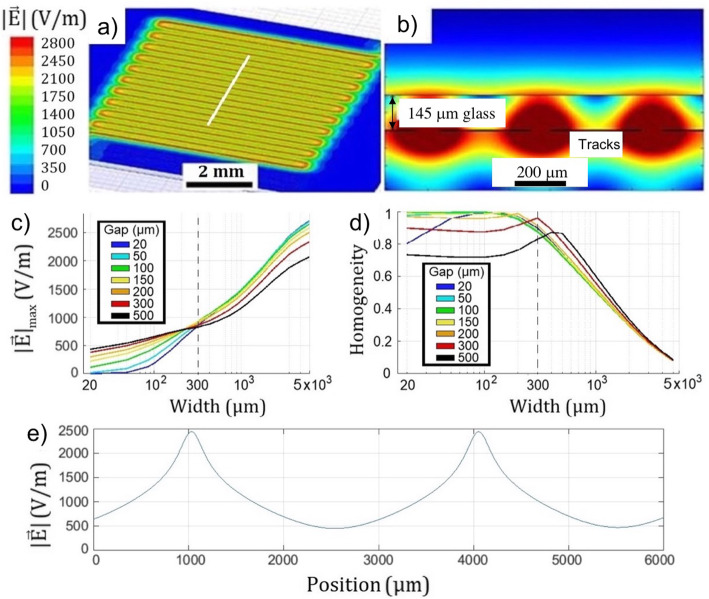
**a** Simulation of the intensity of an electrical field over a 145 μm glass placed above a circuit with width tracks of 225 μm and a gap of 50 μm. The white lines indicate the cross section shown in part b. **b** cross section of the electric field. **c** Peak value of the electrical field along the glass surface depending on the width and separation of the tracks and **d** Homogeneity of the electrical field depending on the width and separation of the tracks. **e** Electrical field along the surface of the glass for a circuit with 3 mm width tracks and 20 μm gap

To study the shape and intensity of the electrical field generated by the circuit, simulations were performed using the software ANSYS Maxwell. Simulations were performed applying a voltage of 5 V between the terminals of different models. In each case, results show an electrical field fluctuating spatially between two values, with a period equal to the distance between the center of two consecutive tracks. This behavior can be observed in Fig. 3 a and b.

Figure 3a shows the intensity of the electric field induced on the surface of a 145 μm glass (microscope cover slip) by an aluminum circuit placed underneath. In this figure, a circuit with track width of 225 μm and a gap of 50 μm between tracks was simulated. A cross section of the electrical field propagating through the thin glass is shown in Fig. 3b. Note the oscillatory behavior of the electrical field profile at the interface glass-air in the orthogonal direction to the tracks. As consequence of this behavior, it is necessary to define the homogeneity of the electrical field as

6 $$ H=\frac{{\left|\overrightarrow{E}\right|}_{min}}{{\left|\overrightarrow{E}\right|}_{max}} $$

According eq. (6), the electric field will be more homogeneous when the value of *H* approaches 1, while the oscillations will be more relevant the closer the value of h approaches zero. The electrical field induced by the circuit along the direction perpendicular to the circuit tracks (represented with a white line in Fig. 3a) was characterized for tracks widths in a range from 20 to 5000 μm and for gaps between them from 20 to 500 μm. Results of the peak value of the electrical field are shown in Fig. 3c, and Fig. 3d shows the homogeneity of the electrical field for the same circuit geometries. Simulations show that electrical field amplitude increases with the width of the tracks while the homogeneity of the field over the cover slip decreases. On the other hand, in Fig. 3c two regions are clearly differentiated. If the tracks width is less than 300 μm, those circuits with a greater gap between tracks will provide a greater field. On the contrary, when the width of the tracks exceeds 300 μm, the circuit that produces the largest electric field will be the one with the smallest gap between tracks. These two regions are also distinguished in Fig. 3d where it is shown that more homogeneous electric fields are generated for those circuits with less gap between tracks. Furthermore, the homogeneity is almost constant for tracks width less than 200 μm and reaches a maximum depending on the gap between the tracks. An abruptly decrease of the field homogeneity is observed for tracks width between 200 and 500 μm and gaps from 200 to 500 μm.

To study electrical cell stimulation, a low homogeneity electric field was selected. Figure 3e shows the electric field corresponding to a circuit with 3 mm width tracks and 20 μm gap that provides an oscillating electric field between a minimum of 455 V/m and a maximum of 2437 V/m.

Once the manufacturing process was completed, the programming of a control software that allowed to apply different electrical signals to cell cultures was addressed. With this purpose a specific program connected to an I/O device which generate square signals according to the user’s indications was developed.

The hardware selected to provide the signal was the NI USB-6501 portable digital I/O device, from National Instruments. It provides 5 V by default and up to 8.5 mA. For this application, the program was designed to apply a non-symmetrical square waveform with an amplitude of 5 V. The frequency of the signal, the duration of the pulse and the duration of the signal can be tuned by the user. Almost any modern computer is enough to run and manage both the hardware, which only needs a free USB slot, and the software, which no requires having LabVIEW installed.

### Cell stimulation

**Fig. 4:**
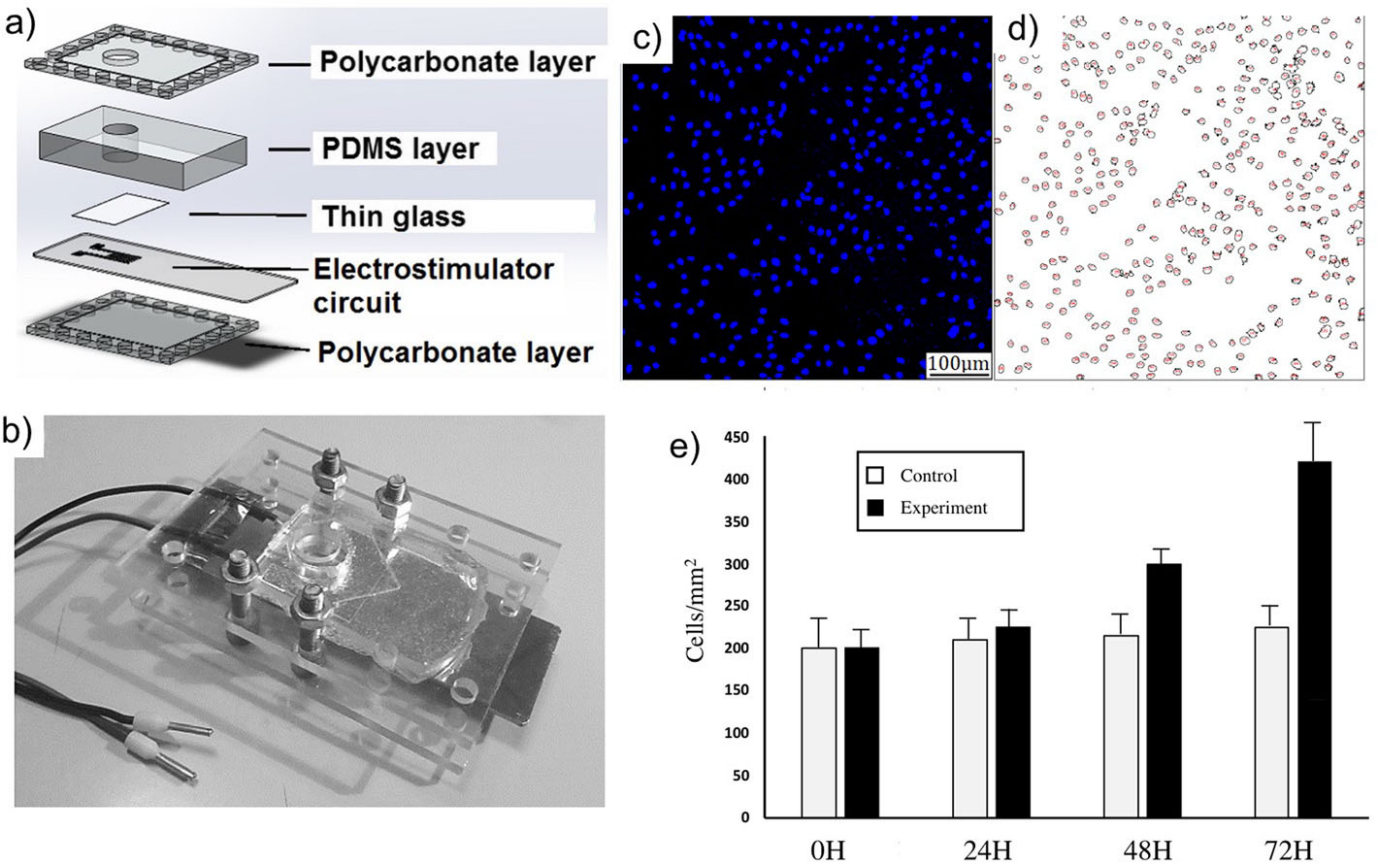
**a** diagram of the electrical stimulator assembled parts, **b** image of the electrical stimulator device, **c** image of a cell culture obtained with a fluorescence microscopy, **d** previous image after being processed with the software ImageJ and **e** Cell density of the samples after a continuous stimulation of 24, 48 and 72 h

Cell Imaging Dishes (Eppendorf, Hamburg, Germany) containing H9c2 cells (50,000 cells were seeded in each cell dish) was placed directly over the electrical stimulator circuit (Fig. 4a and b) and a continuous signal of 5 V was applied, inducing over the surface of the cell imaging dishes the electrical field showed in Fig. 3e. The electrical stimulus (5 V), was applied throughout the experiment for 24, 48 and 72 h. After the experiment, cells were marked with the DAPI fluorescence stain. Fluorescence microscopy images were taken using the Leica TSC SP8 confocal microscope. Cell density was determined by processing the images with the software ImageJ (minimum particle size of 50 pixels and minimum circularity of 0.3).

Figure 4c shows a fluorescence microscopy image (the blue dots are the cell nucleus). Figure 4d shows the same image after being processed by the ImageJ software, which identifies the nucleus for an accurate count. To evaluate the number of cells fluorescence microscopy images with an area of 1.385 mm2 were analyzed (between 4 and 10 depending on the quality of the image for each condition). Results are presented as the mean value of all the images processed and the error as 2σ (the half-width of a 95% confidence interval). Results of the cell density count are shown in Fig. 4e for the samples exposed for 24, 48 and 72 h, as well as the count in the control samples. It can be observed a remarkable increase in the number of cells for the stimulated samples, where after 72 h the cell density doubles the cell density of the control samples. Note that the bovine serum was removed from the culture medium to avoid any effect on the cells so that cells proliferation is due solely to the effect of the electrostimulation.

## Discussion

Electrical signals are known to play an important role during cardiac tissue development. Recent “in vitro” studies have shown that low voltage electrical stimulation (ES) plays an important role in regulating cell, differentiation, proliferation adhesion, matrix formation, and migration [36–38].

Cell alignment and elongation in the direction of electrical stimulation are commonly observed after culturing cardiomyocytes in an electrical stimulation field [39]. To achieve greater cardiac cell maturation and function “in vitro”, some efforts has been made to understand the role of mechanical and electrical stimulation in cardiac cell gene and protein expression. There has been a significant amount of effort in development of culture platforms that improve cardiac function compared to traditional 2D culture, where cells do not align and remain relatively immature [40]. The application of electrical stimulation to cells culture to influence cell proliferation has been also investigated as a possible method to increase “in vitro” maturation of cardiac cells [4, 5]. In this sense, electric biochips where to apply electrical stimulus to cells reveals as a powerful tool for 2D and 3D culture.

Nevertheless, commonly used electrical stimulators present the same assembly that consist of two electrodes which are directly in contact with cells and culture medium which is not appropriate because the electrodes may cause toxic reactions in the culture or may be degraded by electrochemical reactions with the cell medium. To prevent such reactions, some of then uses salt bridges submerged in the culture media to stimulate cells. These salt bridges are used to separate cells from metallic electrodes which prevents media pH modifications and the generation of electrochemical products.

These type of chambers, are not easy to operate by the following reasons: 1) cell exposure time to the electrical stimulation is limited due the concentration and heat differences between the bridge contents and the media drives the diffusion of salt and temperature into the media and vice-versa, 2) it is difficult to run several chambers simultaneously, 3) it is challenging to maintain sterility, 4) the working area is small. Furthermore, devices used to deliver DC electrical stimulation in the clinical setting use metallic electrodes rather than salt bridges, thus making results obtained during in vitro and in vivo experiments difficult to correlate [41]. To solve these issues, the laser based fabrication process that we have used for fabricating the electrical tracks of the bioelectronics-on-a-chip device is relatively accessible, flexible (in terms geometries and sizes with possibility of fabricating multiple chambers systems) and low cost, compared with another similar device fabricated by photolithography and chemical baths. Cardiac tissue engineering has grown in last decades in parallel with the development of human cell in vitro constructs. Nevertheless, the functionality maintenance of cardiac tissue has not being achieved. Bioelectronics tissue platforms where to apply electrical and mechanical stimulation of tissues can promote the cardiomiogenesis in vitro by mimicking the complexity of the in vivo microenvironment. The results presented in this work shows a remarkable increase in the number of H9c2 cells for the stimulated samples. The electrical field stimulation device presented here enables researchers to expose cells to ES with an easy to use, re-usable, adjustable, and inexpensive chamber and envisage the potential applications on electrophysiology studies, monitoring and modulate cellular behavior through the application of electric fields.

## Conclusions

Cell proliferation of Rat ventricular cardiomyoblasts cells (H9c2) using the bioelectronics-on-a-chip was enhanced upon the electrical stimulation. It can be observed a remarkable increase in the number of cells for the stimulated samples, where after 72 h the cell density doubles the cell density of the control samples.

## Data Availability

All data generated or analyzed during this study are included in this published article.

## Abbreviations

PDMS: Polydimethylsiloxane
H9c2: Rat ventricular cardiomyoblasts cells
ES: Electrical stimulation
PVD: Physical vapor deposition
FBS: Fetal bovine serum
BSA: Bovine serum albumin
DAPI: 4′,6-diamidino-2-phenylindole
EHT: Extra high tension
SEM: Scanning Electron Microscope
DMEM: Dulbecco’s Modified Eagle *Medium*
